# Prognostic significance of plasma IL-2 and sIL-2Rα in patients with first-ever ischaemic stroke

**DOI:** 10.1186/s12974-020-01920-3

**Published:** 2020-08-14

**Authors:** Haiping Zhao, Fangfang Li, Yuyou Huang, Sijia Zhang, Lingzhi Li, Zhenhong Yang, Rongliang Wang, Zhen Tao, Ziping Han, Junfen Fan, Yangmin Zheng, Qingfeng Ma, Yumin Luo

**Affiliations:** 1 Cerebrovascular Diseases Research Institute and Department of Neurology, Xuanwu Hospital of Capital Medical University, 45 Changchun Street, Beijing, China; 2 National Clinical Research Center for Geriatric Disorders, Beijing, China; 3 Beijing Institute for Brain Disorders, Beijing, China

**Keywords:** Acute ischaemic stroke, Lymphocyte, sIL-2Rα, IL-2, Prognosis

## Abstract

**Background:**

An imbalance between circulating neuroprotective and neurotoxic T cell subsets leads to poor prognosis in acute ischaemic stroke (AIS). Preclinical studies have indicated that the soluble form of the interleukin-2 receptor α (sIL-2Rα)-IL-2 complex regulates T cell differentiation. However, the association between sIL-2Rα levels and AIS remains unclear.

**Methods:**

A total of 201 first-ever AIS patients within 24 h after stroke onset and 76 control subjects were recruited. The National Institutes of Health Stroke Scale (NIHSS) score and 3-month functional outcome (modified Rankin Scale [mRS] score) at admission were assessed. Plasma sIL-2Rα and IL-2 levels at admission were measured. Prognostic significance was identified by using univariate and multivariate logistic regression analyses.

**Results:**

Patients with poor functional outcomes at 3 months had significantly higher levels of sIL-2Rα and lower levels of IL-2 than patients with good outcomes. Moreover, sIL-2Rα levels showed a strong positive correlation with NIHSS and mRS scores (*p* < 0.0001), whereas IL-2 levels were negatively correlated with mRS scores (*p* < 0.01). Univariate analyses showed that higher sIL-2Rα and IL-2 levels were associated with an increased and reduced risk of unfavourable outcomes, respectively. After adjusting for confounding variables, the sIL-2Rα level remained independently associated with an increased risk of an unfavourable outcome, and adding sIL-2Rα levels to the conventional risk factor model significantly improved risk reclassification (net reclassification improvement 17.56%, *p* = 0.003; integrated discrimination improvement 5.78%, *p =* 0.0003).

**Conclusions:**

sIL-2Rα levels represent a novel, independent prognostic marker that can improve the currently used risk stratification of AIS patients. Our findings also highlight that elevated plasma sIL-2Rα and IL-2 levels manifested opposite correlations with functional outcome, underlining the importance of IL-2/IL-2R autocrine loops in AIS.

## Background

Stroke is a major cause of global disease burden with limited therapies [1]. One of the many challenges is to identify a cost-effective diagnostic or prognostic biomarker for acute ischaemic stroke (AIS). Increasing evidence has pointed out that an imbalance between circulating neuroprotective and neurotoxic T lymphocyte subsets, such as regulatory T (Treg) and T-helper 17 (Th17) cells, may contribute to poor prognosis in AIS patients [2, 3]. Although various strategies to expand the number of Tregs and inhibit the activation of effector T cells have yielded successful results in preclinical studies [4, 5], the cause of the imbalance in human T cells after AIS has not been well investigated.

Interleukin-2 (IL-2) functions as a multilineage lymphocyte growth factor to promote the proliferation and differentiation of CD4+ T cells into Th1, Th2, Th17 and Treg subsets by binding to low-, medium- or high-affinity receptors (IL-2Rα/IL-2Rβ/γc). In recent years, IL-2 was shown to have therapeutic efficacy by expanding distinct T cell compartments, since low-dose IL-2 preferentially expands Tregs versus other immune cells [6], and some Treg-biased anti-IL-2 antibodies (IL-2Ab) have been developed as therapies for immune diseases [7]. Subsequent work has validated the therapeutic applications of IL-2/IL-2Ab in preclinical models of cerebral ischaemia by inducing Treg proliferation [4]. Although IL-2 is one of the most frequently studied cytokines in AIS patients, the conclusions are ambiguous and inconsistent with the results of experimental studies due to the extensive functionality of IL-2. Thus, more detailed insight is needed into clinically feasible immune therapy targeting the IL-2/IL-2R system in AIS patients.

Elevated plasma levels of the soluble form of interleukin-2 receptor α (sIL-2Rα) reflect the inflammatory process with enhanced T cell activation. Elevated serum levels of sIL-2Rα have been correlated with a poor prognosis in a variety of different types of cancers, immune diseases, and cardiovascular events, including stroke and mortality [8–10]. By binding to IL-2, sIL-2Rα upregulates Foxp3 expression and induces the development of regulatory T (Treg) cells [11]. However, its biological relevance in clinical studies remains unclear and controversial. In addition, the association between the plasma level of sIL-2Rα and ischaemic stroke outcome has not been investigated. Therefore, we undertook the present study to explore the clinical significance of sIL-2Rα levels in AIS compared to those of IL-2 to provide new insight into the potential function of IL-2/IL-2R autocrine loops in AIS patients.

## Methods

### Study participants

**Fig. 1:**
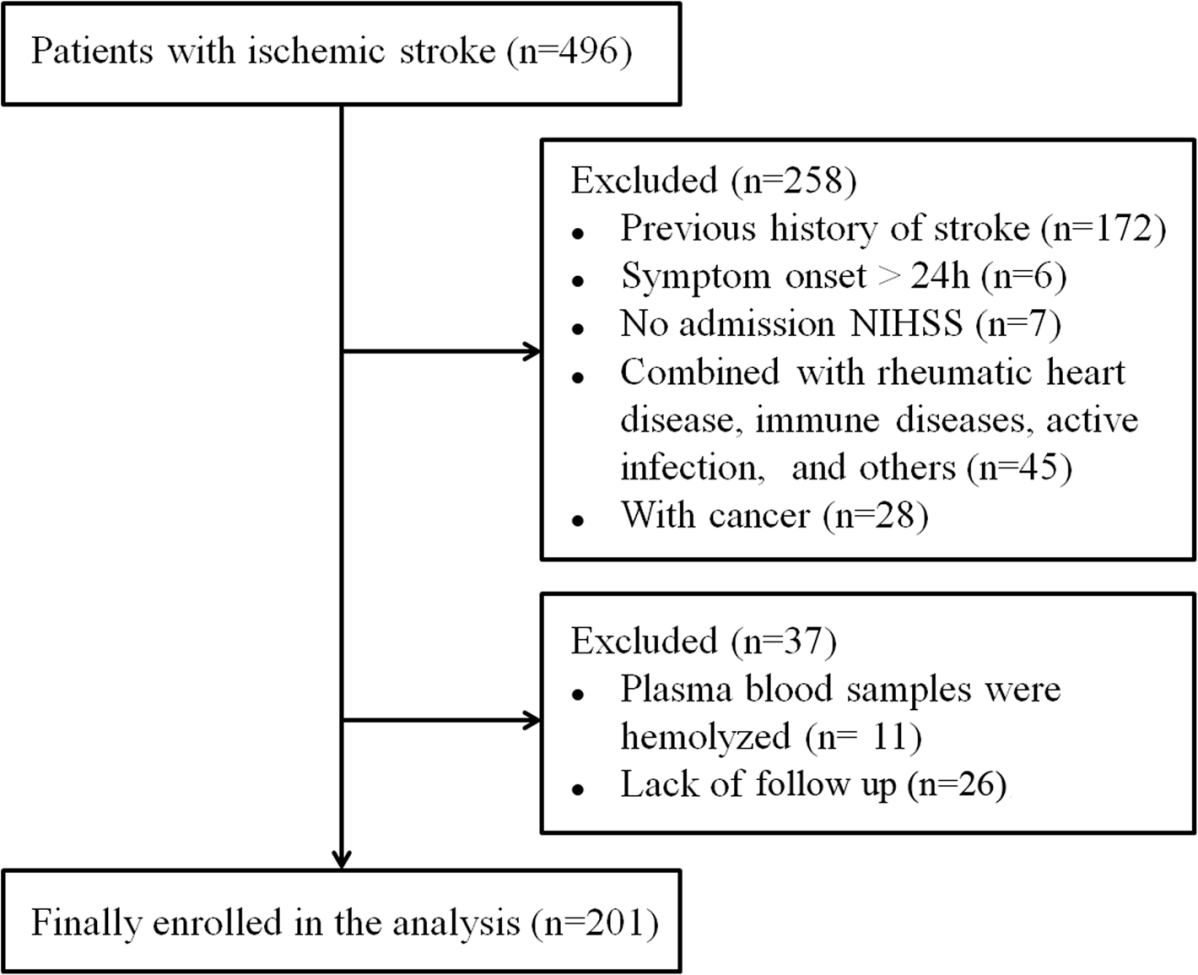
Study flow chart

The data that support the findings of this study are available from the corresponding author upon reasonable request. We retrospectively screened a consecutive range of patients who were diagnosed with first-ever AIS and presented to Xuanwu Hospital of Capital Medical University within 24 h after symptom onset between November 2018 and May 2019. Our study was approved by the Ethics Committee of Xuanwu Hospital, Capital Medical University. All patients or their immediate family members provided written informed consent. The inclusion criteria were (1) focal or global neurological deficits, (2) a diagnosis of AIS confirmed by brain magnetic resonance imaging (MRI) or computed tomography (CT), (3) no history of stroke, and (4) no pre-stroke disability. Patients with cerebral haemorrhage within the previous 3 months, cancer, rheumatic heart disease, heart failure, renal failure, liver cirrhosis, immune diseases, active infection, epilepsy or other neurological diseases were excluded from our study. Finally, 201 AIS patients were enrolled in our analysis (Fig. 1). Healthy controls were age- and sex-matched relatives of the AIS patients, and those who were examined by experienced neurologists and found to be free of cerebrovascular diseases for more than 12 months were recruited.

### Clinical data and blood collection

Baseline data, including demographic characteristics, onset time, blood pressure, comorbidities, and routine laboratory determinations (neutrophil number, lymphocyte number, plasma glucose levels, blood lipids, etc.) at admission were collected from all study participants. Recanalization treatment included recombinant tissue-type plasminogen activator treatment and endovascular treatment. Stroke severity was assessed using the National Institutes of Health Stroke Scale (NIHSS) at admission by trained neurologists. Clinical outcome was evaluated 90 days after stroke by experienced neurologists who were blinded to the biomarker levels using the modified Rankin Scale (mRS). We defined a favourable outcome as a mRS score of 0–2 and an unfavourable outcome as a mRS score of 3–6. Blood samples were collected into K3 EDTA tubes from each AIS patient and healthy control before any treatment. All plasma samples were separated and frozen at − 80 °C.

### Measurements of plasma sIL-2Rα and IL-2 levels

Plasma levels of sIL-2Rα and IL-2 were measured in 201 AIS patients and 76 controls using ProcartaPlex™ Multiplex Immunoassay (eBioscience) according to the manufacturer’s instructions [12]. Supernatant samples were then thawed and clarified by centrifugation at 10,000 × *g* for 10 min. Clarified samples were stored on ice until they were loaded onto plates (∼1 h). Wash buffer (10×) was diluted in deionised water to make a 1× solution, and 1 vial of lyophilized Standard Mix containing all 10 proteins was reconstituted with 250 μL of the same media used to generate the samples. The standard vial was vortexed briefly, centrifuged gently for 10 s and stored on ice for 10 min. A 1:4 serial dilution was performed for a 7-pt standard curve with varying S1–S7 concentrations for each target (see Table 1) and a background tube. Bead Mix (1×) was vortexed for 30 s, and 50 μL was added to each assay well for standards, background and samples. For the wash steps, a magnetic plate separator (Thermo Fisher Scientific Cat. No. EPX-55555-000) was used. Beads were allowed to settle for 2 min, and then the liquid was decanted with a manual pipette. Then, 150 μL of wash buffer was added to each well with beads and allowed to incubate for an additional 15–30 s. The liquid was decanted, and the plate was blotted gently on absorbent paper to remove excess wash buffer. Then, the plate was removed from the magnetic separator. Fifty microlitres of standards, background and samples were run in duplicate. The plate was sealed, and a plate cover was added to protect the plate from light. The plate was loaded onto a small diameter (< 5 mm) plate shaker for 1 min at 800 revolutions per minute (rpm) and then adjusted to 600 rpm for 2 h at room temperature. Wash steps were performed as described above. After the wash steps, 25 μL of detection antibody (1×) was added to each well. The plate was sealed, covered and shaken for 30 min at room temperature. Wash steps were performed as described above. After the wash steps, 50 μL of streptavidin-PE was added to each assay well. The plate was sealed, covered and shaken for 30 min at room temperature. Wash steps were performed as described above. After the wash step, 120 μL of Reading Buffer was added to each assay well. The plate was sealed, covered and shaken at 800 rpm for 5 min at room temperature. Then, the plate cover and seal were removed, and the plate was loaded into the Luminex 200 (LX200) system for reading, which took approximately 15 min. The LX200 with xPonent® 3.1 was allowed to warm for a minimum of 30 min. Calibration and verification steps were performed per the manufacturer’s recommendations. The xPonent software was programmed using the settings from the assay protocol and certificate of analysis. These assays were performed by experienced laboratory technicians blinded to the treatments and conditions.

**Table 1:** Comparison of cytokine levels between acute ischaemic stroke patients and healthy controls

Characteristic	Control (*n* = 76)	AIS (*n* = 201)	*p* value
Age, year; mean ± SD	62.63 ± 11.38	64.11 ± 13.29	0.237
Male, *n* (%)	51 (67.11)	149 (74.13)	0.244
Inflammatory cytokines, median (IQR), pg/ml			
sIL-2Rα	812.80 (612.91–1170.57)	947.63 (689.61–1370.90)	0.036
IL-2	21.20 (12.94–32.77)	28.08 (17.28–40.85)	0.002

*IL-2* interleukin-2, *sIL-2Rα* soluble interleukin-2 receptor α

### Statistical analysis

Data were analysed with SPSS 21.0 software (IBM Corp., Armonk, NY, USA) and R software (version 3.5.1). Statistical significance was set at *p* < 0.05. Continuous variables with a normal distribution were expressed as the means ± SDs and analysed using Student’s *t* test. Non-normally distributed variables were expressed as medians with interquartile ranges (IQRs) and analysed using the Mann-Whitney *U* test. For correlation analyses, the Spearman correlation coefficient (rho) was calculated. The chi-squared test was used to compare the frequencies and percentages of categorical variables. Univariate and multivariable logistic regression analyses were used to analyse the relationship between cytokines and the 3-month functional outcomes of AIS patients. The crude and adjusted odds ratios (ORs) and 95% confidence intervals (CIs) of each biomarker were calculated. The multivariable logistic regression analysis included variables that either were previously reported or associated with stroke outcomes in the univariate logistic analyses (*p* < 0.10). Furthermore, we calculated the net reclassification improvement (NRI) and integrated discrimination improvement (IDI) to quantify the improvement in the correct reclassification and sensitivity with the addition of plasma sIL-2Rα levels to the established risk model according to previous literature [13].

## Results

### Elevated sIL-2Rα levels were correlated with an increased risk of adverse outcomes of AIS

**Fig. 2:**
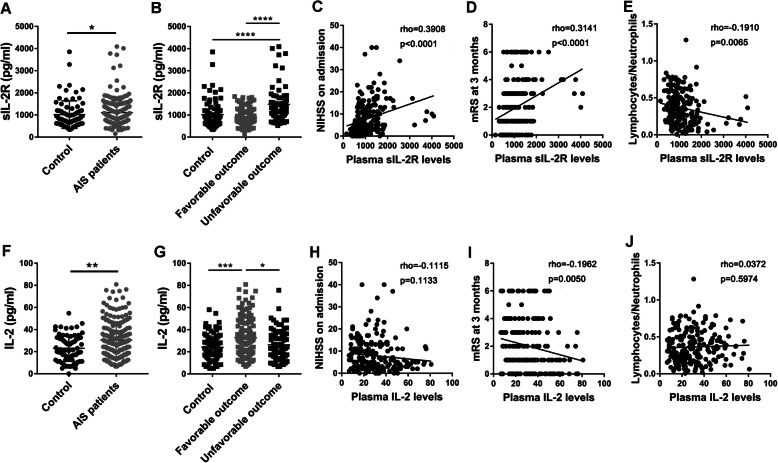
Changes in plasma levels of sIL-2Rα and IL-2 in AIS patients and their correlation with neurological deficits. **a** Plasma level of sIL-2Rα detected by ProcartaPlex in AIS patients and control subjects. **b** Plasma level of sIL-2Rα in AIS patients with favourable (mRS score = 0–2) and unfavourable (mRS score = 3–6) functional outcomes. **c**–**e** Correlation of admission NIHSS score, 3-month mRS score and lymphocyte/neutrophil ratio with the plasma level of sIL-2Rα. **f** Plasma levels of IL-2 detected by ProcartaPlex in AIS patients and control subjects. **g** Plasma levels of IL-2 in AIS patients with favourable (mRS score = 0–2) and unfavourable (mRS score = 3–6) functional outcomes. **h**–**j** Correlation of admission NIHSS score, 3-month mRS score and lymphocyte/neutrophil ratio with the plasma level of IL-2; *N* = 76 for controls, *N* = 201 for AIS patients (*N* = 128 for the favourable outcome group, *N* = 73 for the unfavourable outcome group). ****p < 0.0001, ***p < 0.001, *p < 0.05. AIS, acute ischaemic stroke; sIL-2Rα, soluble interleukin-2 receptor α; IL-2, interleukin-2; NIHSS, National Institutes of Health Stroke Scale; mRS, modified Rankin Scale

Plasma sIL-2Rα levels were significantly increased in AIS patients compared to control subjects (*p* < 0.05, Fig. 2a, Table 1), and a subgroup analysis of patients based on favourable (mRS score 0-2) and unfavourable prognosis (mRS score 3-6) revealed that sIL-2Rα levels were markedly higher in AIS patients with adverse outcomes than in those with good outcomes (*p* < 0.0001) or control subjects (*p* < 0.0001, Fig. 2b, Table 1). Furthermore, correlation analysis showed that sIL-2Rα levels were prominently positively correlated with the NIHSS score at admission (*p* < 0.0001, Fig. 2c) and the mRS score at 3 months post-stroke (*p* < 0.0001, Fig. 2d) and were significantly negatively correlated with the lymphocyte/neutrophil ratio (*p* < 0.01, Fig. 2e). Baseline characteristics of the 201 AIS patients stratified by functional outcome (mRS score) at 3 months are shown (Table 2). Poor prognosis was found in 71 (35.32%) patients, with 50 of them (70.42%) being male, and mainly occurred in older patients and patients with high NIHSS scores and glucose levels, a history of atrial fibrillation, high neutrophil-to-lymphocyte ratios and low triglyceride and total cholesterol levels.

**Table 2:** Baseline characteristics of AIS patients with favourable and unfavourable outcomes

Baseline characteristics	All (201)	Favourable outcome (130)	Unfavourable outcome (71)	*p* value
Age, year	64.11 ± 13.29	61.86 ± 12.33	68.23 ± 14.07	0.001
Male, *n* (%)	149 (74.13)	99 (76.15)	50 (70.42)	0.375
Time from onset, h	3.00 (1.50–5.10)	2.90 (1.30–4.55)	3.20 (1.80–6.80)	0.086
Baseline systolic BP, mmHg	150 (140–168.5)	152 (140–170)	148 (138–168)	0.206
Baseline diastolic BP, mmHg	87.0 (78.0–93.0)	85.0 (77.8–93.3)	90.0 (79.0–92.0)	0.875
Baseline NIHSS score	5.0 (3.0–11.0)	4.0 (2.0–6.0)	13.0 (9–17.0)	0.000
Recanalization treatment	103 (51.24)	74 (56.92)	29 (40.85)	0.029
Risk factors, *n* (%)				
Hypertension	132 (65.67)	82 (63.08)	50 (70.42)	0.294
Diabetes mellitus	82 (40.80)	47 (36.15)	35 (49.30)	0.070
Coronary heart disease	46 (22.89)	24 (18.46)	22 (30.90)	0.043
Atrial fibrillation	32 (15.92)	11 (8.46)	21 (29.58)	0.000
Stroke aetiology, *n* (%)				
Thrombotic	147 (73.13)	94 (72.31)	53 (74.65)	0.721
Embolic	15 (7.46)	10 (7.69)	5 (7.04)	0.867
Lacunar	39 (19.40)	26 (20.00)	13 (18.31)	0.772
Clinical parameters, median (IQR)				
Neutrophil-to-lymphocyte ratio	2.90 (2.03–5.25)	2.55 (1.80–4.07)	4.53 (2.35–9.08)	0.000
Baseline glucose, mmol/L	6.77 (5.71–8.94)	6.33 (5.56–8.04)	8.30 (6.47–10.54)	0.000
Triglycerides, mmol/L	1.45 (0.96–2.47)	1.70 (1.0–-2.69)	1.20 (0.76–1.72)	0.002
Total cholesterol, mmol/L	4.53 (3.79–5.43)	4.69 (3.93–5.59)	4.26 (3.60–5.10)	0.008
High-density lipoprotein, mmol/L	1.18 (1.00–1.39)	1.17 (0.97–1.39)	1.23 (1.05–1.37)	0.373
Low-density lipoprotein, mmol/L	2.70 (2.03–3.42)	2.79 (2.05–3.62)	2.45 (1.99–3.11)	0.069
Biomarkers (pg/ml), median (IQR)				
sIL-2Rα	947.63 (689.61–1370.90)	812.92 (631.42–1160.78)	1331.25 (947.63–1657.90)	0.000
IL-2	28.08 (17.28–40.85)	29.53 (18.67–46.19)	24.29 (15.73–36.60)	0.027

*BP* blood pressure, *NIHSS* NIH Stroke Scale, *IQR* interquartile range

### Increased IL-2 levels were correlated with a reduced risk of unfavourable outcomes of AIS

Plasma IL-2 levels were also markedly upregulated in AIS patients compared to control subjects (*p* < 0.01, Fig. 2f, Table 1). Subgroup analysis revealed that in contrast to sIL-2Rα levels, IL-2 levels were higher in AIS patients with favourable outcomes than in those with unfavourable outcomes (*p* < 0.05) or control subjects (*p* < 0.001, Fig. 2g). Moreover, IL-2 levels showed no correlation with NIHSS scores (Fig. 2h) or lymphocyte/neutrophil ratios (Fig. 2j) but were negatively correlated with mRS scores at 3 months post-stroke (*p* < 0.05, Fig. 2i).

### sIL-2Rα levels represented an independent predictor for unfavourable outcomes of AIS

We further examined the predictive value of sIL-2Rα and IL-2 levels for unfavourable outcomes after AIS and found that in the univariate analyses, high levels of sIL-2Rα were associated with an increased risk of unfavourable outcomes (*p* < 0.0001, Table 2), while high levels of IL-2 were associated with a decreased risk of unfavourable outcomes (*p* < 0.05, Table 2).

After adjusting for age, admission NIHSS score, a history of diabetes mellitus, coronary heart disease and other variables in the binominal multivariate logistic analysis (model 2), sIL-2Rα levels remained significant for the prediction of unfavourable outcome in AIS patients (*p* < 0.0001, Table 3). The multivariable adjusted OR (95% CIs) for sIL-2Rα levels (each 100 pg/ml increase) was 1.197 (1.085–1.322). Based on the receiver operating characteristic curve, the optimal cut-off value of the plasma sIL-2Rα level as an indicator for unfavourable outcome was 971.44 pg/ml, yielding a sensitivity of 74.6% and a specificity of 66.9%, with an area under the curve of 0.760. Then, sIL-2Rα levels were dichotomized using the cut-off point identified by the receiver operating characteristic curve. Consistent with the result that the sIL-2Rα level was regarded as a continuous variable, high sIL-2Rα levels (sIL-2Rα ≥ 971.44 pg/ml) remained associated with an increased risk of poor outcome at 3 months after AIS (OR, 5.577; 95% CI, 2.264–13.740). However, we did not find that IL-2 levels were independently associated with unfavourable outcomes after adjusting for confounding variables (Table 3).

**Table 3:** Biomarkers and the risk of the primary outcome after AIS

	Model 1		Model 2	
	OR (95% CI)	*p* value	OR (95% CI)	*p* value
Biomarkers (as continuous variables)				
sIL-2Rα, per 100 pg/ml increase	1.235 (1.142–1.335)	0.000	1.197 (1.085–1.322)	0.000
IL-2, per 1 pg/ml increase	0.976 (0.957–0.995)	0.013	0.991 (0.965–1.017)	0.400
Biomarkers (as categorical variables)				
sIL-2Rα, ≥ 971.44 pg/ml	5.957 (3.117–11.384)	0.000	5.577 (2.264–13.740)	0.000

Model 1 was an unadjusted logistic regression model. The variables in model 2 included age, admission NIHSS score, a history of diabetes mellitus, coronary heart disease, atrial fibrillation, recanalization treatment, neutrophil-to-lymphocyte ratio, glucose, total cholesterol, low-density lipoprotein, sIL-2Rα and IL-2 levels on admission

### Incremental predictive value of sIL-2Rα levels for AIS

We further investigated whether the addition of plasma sIL-2Rα levels to a conventional model with established risk factors could improve its predictive power for unfavourable outcomes (Table 4). The results showed that the addition of plasma sIL-2Rα levels to the conventional model improved the NRI by 17.56% (*p* = 0.003) and the IDI by 5.78% (*p* = 0.0003) for the prediction of unfavourable outcomes.

**Table 4:** Reclassification of the primary outcome by plasma cytokine levels among AIS patients

Models	NRI		IDI	
	Estimate (95% CI), %	*p* value	Estimate (95% CI), %	*p* value
Conventional model	Reference	–	Reference	–
Conventional model + sIL-2Rα	17.56 (5.98–29.15)	0.003	5.78 (2.64–8.93)	0.0003

*Abbreviations*: *CI* confidence interval, *IDI* integrated discrimination index, *NRI* net reclassification improvement. Risk factors included in the conventional model were sex, recanalization treatment and NIHSS score on admission

## Discussion

We found for the first time that increased levels of sIL-2Rα might be associated with unfavourable outcomes at 3 months after AIS. Particularly, the addition of plasma sIL-2Rα levels to a conventional model with established risk factors substantially improved the risk stratification for primary outcomes. In addition, elevated plasma sIL-2Rα and IL-2 were positively and negatively associated with unfavourable functional outcomes in AIS patients, respectively, indicating that sIL-2Rα and IL-2 might play antagonistic roles in AIS development and that sIL-2Rα is a more important therapeutic target for AIS.

Stroke has high mortality and morbidity. Moreover, biomarkers are required to predict stroke outcomes, which could help clinicians provide rational approaches for patient management. To date, a number of studies have demonstrated that the blood levels of cytokines, chemokines, and growth factors, including C-reactive protein, TNF-α, IL-6 and IL-10, are associated with outcomes in AIS patients [14–16]. Although IL-2 is one of the most frequently studied cytokines in stroke patients, the conclusions are ambiguous and inconsistent with the results of experimental studies [17–19]. This might be due to the complexity of IL-2/IL-2R autocrine loops. IL-2R is composed of IL-2Rα (CD25), IL-2Rβ (CD122) and IL-2Rγ (CD132) subunits and exists as either αβγ heterotrimeric or βγ heterodimeric complexes. When T cells are activated, the IL2-Rα subunit is released into the blood and becomes sIL-2Rα. It has been reported that sIL-2Rα levels were significantly increased in ischaemic left ventricular dysfunction patients [20, 21] and were positively associated with internal carotid wall thickness and cardiovascular disease mortality [10]. Similarly, we found for the first time that plasma sIL-2Rα levels were significantly increased in AIS patients within 24 h after stroke attack and that sIL-2Rα levels were markedly higher in AIS patients with adverse outcomes than in those with favourable outcomes. In contrast to sIL-2Rα levels, IL-2 levels were higher in AIS patients with favourable outcomes than in those with poor outcomes. Furthermore, univariate analyses confirmed this conclusion, demonstrating that elevated plasma sIL-2Rα and IL-2 levels were associated with an increased and decreased risk of unfavourable outcomes at 3 months, respectively. However, after adjusting for potential contributing factors in the binominal multivariate analysis, we showed that sIL-2Rα levels represented an independent biomarker for predicting functional outcomes in AIS patients and were superior to IL-2 levels. Furthermore, the addition of plasma sIL-2Rα levels to conventional risk factors was shown to improve risk predictions for the primary outcome. We first showed that plasma sIL-2Rα levels might represent a new independent biomarker in AIS risk stratification, which could be beneficial for selecting high-risk patients to receive more aggressive monitoring and therapeutic interventions in future clinical practice.

In numerous clinical observations, the proportion of circulating immune cells is critical to the neurological prognosis of AIS patients, and a low ratio of lymphocytes was shown to predict poor functional outcomes after AIS [22]. We noticed a significant negative correlation between sIL-2Rα levels and lymphocyte/neutrophil ratios, indicating that sIL-2Rα levels might affect the proliferation of human lymphocytes after AIS. With the exception of total lymphocytes, the imbalance between circulating neuroprotective and neurotoxic T lymphocyte subsets also affects prognosis in AIS. Decreasing circulating Tregs and increasing Th17 has been shown to contribute to poor prognosis in AIS [23–29]. IL-2 functions as a multilineage lymphocyte growth factor to promote the proliferation and differentiation of naive CD4+ T cells. The IL2-Rα subunit is constitutively expressed at high levels on regulatory T (Treg) cells and at lower levels on natural killer cells and resting effector CD8^+^ T cells, resulting in differential IL-2 potency between the different immune cell compartments. However, when T cells are activated, the IL2-Rα subunit is released into the blood and becomes sIL-2Rα. Preclinical studies indicated that sIL-2Rα competes with IL-2 for binding to IL-2R at the T cell surface and disturbs T cell differentiation [30]. According to our data, increased sIL-2Rα and IL-2 levels were associated with an increased and reduced risk of unfavourable outcomes, respectively. Thus, we speculate that abnormal upregulation of sIL-2Rα is involved in the imbalance between neuroprotective CD4^+^ T cell subsets (Th2 and Treg) and neurotoxic CD4^+^ T cell subsets (Th1 and Th17) and promotes neuroinflammation and poor prognosis after AIS. Above all, it is of interest to further elucidate the mechanisms by which the IL2-Rα subunit is released into the blood and increased sIL-2Rα or IL-2/IL-2Rα autocrine loops affect the proportion of CD4^+^ T cell subsets in AIS.

## Conclusions

Our findings provide important clues for both clinical application and basic study. Plasma sIL-2Rα levels represent a novel, independent prognostic marker that can improve the currently used risk stratification in AIS patients within 24 h after stroke attack. Therefore, a combination of plasma sIL-2Rα level determination in conjunction with clinical and imaging examinations will yield the greatest accuracy for predicting a poor outcome for AIS. Further studies with larger sample sizes are needed to verify our findings, and further studies including patients with pre-stroke disability as well as a second or subsequent stroke are needed to determine whether sIL-2Rα levels can predict stroke outcome in a broader AIS patient population. The present study also revealed insights into the initial inflammatory response to ischaemic stroke within 24 h and recognised the potential pathogenic function of IL-2/IL-2R autocrine loops in T lymphocyte differentiation after AIS.

## Data Availability

The datasets used during the current study are available from the corresponding author on reasonable request.

## Abbreviations

IL-2: Interleukin-2
sIL-2Rα: Soluble form of interleukin-2 receptor α
mRS: Modified Rankin Scale
NIHSS: National Institutes of Health Stroke Scale
IDI: Integrated discrimination improvement
NRI: Net reclassification improvement
